# Molecular mechanism of endophytic bacteria DX120E regulating polyamine metabolism and promoting plant growth in sugarcane

**DOI:** 10.3389/fpls.2024.1334907

**Published:** 2024-02-27

**Authors:** Ying Qin, Qaisar Khan, Jia-Wei Yan, Yu-Yi Wang, Yang-Fei Pan, Ying Huang, Jiang-Lu Wei, Dao-Jun Guo, Yang-Rui Li, Deng-Feng Dong, Yong-Xiu Xing

**Affiliations:** ^1^ College of Agriculture, Guangxi University, Nanning, China; ^2^ Ecology College, Lishui University, Lishui, China; ^3^ Centre for Biotechnology Research, Guangxi South Subtropical Agricultural Science Research Institute, Chongzuo, China; ^4^ College of Life Sciences and Engineering, Hexi University, Zhangye, China; ^5^ Guangxi Key Laboratory of Sugarcane Genetic Improvement, Key Laboratory of Sugarcane Biotechnology and Genetic Improvement (Guangxi), Ministry of Agriculture and Rural Affairs, Sugarcane Research Institute of Guangxi Academy of Agricultural Sciences, Sugarcane Research Center of Chinese Academy of Agricultural Sciences, Nanning, China

**Keywords:** Klebsiella, antioxidative, phytohormone, transcriptomic, interaction

## Abstract

**Introduction:**

Sugarcane endophytic nitrogen-fixing bacterium *Klebsiella variícola* DX120E displayed broad impact on growth, but the exact biological mechanism, especially polyamines (PAs) role, is still meager.

**Methods:**

To reveal this relationship, the content of polyamine oxidase (PAO), PAs, reactive oxygen species (ROS)-scavenging antioxidative enzymes, phytohormones, 1-aminocyclopropane-1-carboxylic synthase (ACS), chlorophyll content, and biomass were determined in sugarcane incubated with the DX120E strain. In addition, expression levels of the genes associated with polyamine metabolism were measured by transcriptomic analysis.

**Results:**

Genomic analysis of *Klebsiella variícola* DX120E revealed that 39 genes were involved in polyamine metabolism, transport, and the strain secrete PAs *in vitro*. Following a 7-day inoculation period, DX120E stimulated an increase in the polyamine oxidase (PAO) enzyme in sugarcane leaves, however, the overall PAs content was reduced. At 15 days, the levels of PAs, ROS-scavenging antioxidative enzymes, and phytohormones showed an upward trend, especially spermidine (Spd), putrescine (Put), catalase (CAT), auxin (IAA), gibberellin (GA), and ACS showed a significant up-regulation. The GO and KEGG enrichment analysis found a total of 73 differentially expressed genes, involving in the cell wall (9), stimulus response (13), peroxidase activity (33), hormone (14) and polyamine metabolism (4).

**Discussion:**

This study demonstrated that endophytic nitrogen-fixing bacteria stimulated polyamine metabolism and phytohormones production in sugarcane plant tissues, resulting in enhanced growth. Dual RNA-seq analyses provided insight into the early-stage interaction between sugarcane seedlings and endophytic bacteria at the transcriptional level. It showed how diverse metabolic processes selectively use distinct molecules to complete the cell functions under present circumstances.

## 1 Introduction

Sugarcane is one of the most important sugar and bioenergy crops in the world (Khan et al., 2021). The increasing population has a higher demand for sugar and bioenergy, which has increased pressure for higher yield and production. To achieve higher yield and production of crops, excessive use of chemical fertilizers, fungicides, pesticides, and herbicides are under application. However, such practices have caused severe environmental damage and deteriorated soil quality. In this regard, exploiting the benefits of interaction between plants and microflora could be an effective approach. Endophytic bacteria that have the ability to colonize the inside the plants tissue is a subclass of plant growth promoting bacteria (PGPB). PGPB commonly known as plant growth-promoting rhizobacteria (PGPR) (Palacios et al., 2014) are bacteria that boost plant development and promote soil bioremediation by secreting a variety of metabolites and hormones (Peng et al., 2021), through nitrogen fixation (Din et al., 2021), and by increasing other nutrients’ bioavailability through mineral solubilization (Poria et al., 2022; Ramasamy and Mahawar, 2023). Diverse endophytic bacteria associated with sugarcane have been reported to affect their growth and development significantly (Xing et al., 2016; Guo et al., 2022). The beneficial impacts of the endophytic bacteria on host plants are usually greater than those provided by many rhizosphere bacteria. They can benefit host plants directly by improving plant nutrient uptake (Guo et al., 2021) and modulating growth and stress-related phytohormones (Nivedita et al., 2020; Cipriano et al., 2021).

Endophytic bacteria spend at least one life cycle inside the tissues without inducing disease symptoms (Pitiwittayakul et al., 2021). Endophytic microbial interactions exist only when a balance between the chemical reactions of colonizing bacteria and the host plant is achieved and maintained over time. These plant bacteria initiate an induced systemic response, improving overall nutrient uptake and increasing phytohormones and antioxidant production. Most plants contain particular metabolites that probably facilitate the entry of endophytic bacteria (Wang et al., 2022). Some bacterial isolates, such as actinobacterial isolates (El-Tarabily et al., 2020), Fungi (Furtado et al., 2019), and *Serratia liquefaciens CL-1* and *Bacillus thuringiensis* X30 (Han et al., 2018) have the capability to secrete PAs. Polyamines (PAs), including spermine (Spm), spermidine (Spd), and putrescine (Put), are small, ubiquitous nitrogenous compounds (Krysenko and Wohlleben, 2022) and involved in many cellular processes, including maintaining cell macromolecular biosynthesis and survival. Di Martino et al. (2013) reported that changes in polyamine concentration are essential for the regulation of polyamine signaling systems during abiotic stress and plant growth promotion. The fluctuations of PAs during interactions between plants and beneficial microorganisms were demonstrated (Bahari Saravi et al., 2022; Li et al., 2022). Therefore, understanding the polyamine metabolism and identification of the associated gene network that enable endophytic bacteria to bring benefits for plants still requires further investigation.

Several transcriptomic studies have revealed the functions of endophytic bacteria during symbiosis between plants and phytopathogens (Ling et al., 2018; Fu et al., 2019; Zhu et al., 2021). These promising results suggest that dual RNA-seq analyses could be used to understand the physiological and molecular mechanism interactions between novel PGPB and crops. Intensive research has contributed to a better understanding of abiotic stress response in crops such as maize (Hardoim et al., 2020), rice (Nivedita et al., 2020), and wheat (Ray et al., 2021). However, the transcriptome-based study of the interactions between plants and PGPB is limited.

*Klebsiella variícola* DX120E (DX120E) is an endophytic bacterium isolated from sugarcane in Daxin County, Guangxi, China (22° 50′ N 107° 11′ E), which has high nitrogenase activity with an excellent capability of phosphorus solubilization, and secreting auxin (IAA) and siderophile (Lin et al., 2015), suggesting its high potential to establish a relationship with host plant (Qin et al., 2022). Özoğul (2004) has detected Spm and Spd by the chromatographic method in *Klebsiella pneumoniae*. *Klebsiella variícola* DX120E owns relative genes involving in polyamine metabolism. However, studies on biogenic amines, interactions and growth promotion mechanisms involving in DX120E-sugarcane systems are unavailable. The current study aimed to promote sugarcane growth by PA producing endophytic plant growth promoting bacteria, including (i) assess the strain’s ability to produce polyamines and promote sugarcane growth under greenhouse conditions; (ii) detect the enzymatic activity of polyamine metabolic pathways, polyamines, ROS-scavenging antioxidative enzymes, and hormone metabolism in micro-plant interactions; and (iii) evaluate the differences in the translation of transcripts in micro plant interaction systems. These results will assist researchers in better understanding the molecular basis of beneficial aspects in host plants and their possible applications as potential inoculants in crop production.

## 2 Materials and methods

### 2.1 Organism

*Klebsiella variícola* DX120E, a bacterial strain isolated from sugarcane variety ROC22 in Guangxi, was cultured on LB solid medium at 37°C, and single colonies were picked for expansion in an LB liquid medium.

### 2.2 Determination of PAs

Bacterial strains were tested for production of Put in Moeller’s decarboxylase agar medium (MDAM) amended with 2 g·L^-1^ of L-arginine-monohydrochloride and phenol red. Plates were incubated at 28°C in the dark for 1 day, and a dark red halo found beneath and around colonies indicated Put production by the decarboxylating isolates (El-Tarabily et al., 2021). Soluble polyamine concentrations in the culture medium were analyzed using the method described by Carolina et al. (2018), adapted to bacterial cultures. The liquid culture supernatant was taken and tested for Spm, Spd, and Put using the ELISA Kit (Jiangsu MEIMIAN Co., China).

### 2.3 Effect of exogenous polyamines on strain growth

Genomic data of DX120E identified multiple genes associated with PA metabolism (Lin et al., 2015). Assays on the effect of exogenous polyamines on the growth of the nitrogen-fixing bacteria DX120E were performed. DX120E was washed 2 times with polyamine-free liquid medium CDM (Li et al., 2019). Media with final concentrations of 0, 0.1, 0.25, 2, 4 and 8 mM of Spm and Spd were prepared. Ten microliters of DX120E at OD_600_ = 1 (concentration of approximately 10^8^ CFU·mL^-1^) were incubated in 10 mL (200 rpm·min^-1^, 28°C) of different concentrations of polyamine medium, and the OD_600_ was measured at different times to observe the growth of the strain by MicroplateReader (Thermo Fisher Scientific Inc., USA).

### 2.4 Effect of DX120E on polyamine metabolism and sugarcane growth

The bacteria were centrifuged at 5000 rpm and 4°C for 10 min, and suspended in sterile distilled water to an OD_600 _ = 1 (concentration of approximately 10^8^ CFU·mL^-1^). The pot experiment was carried out in the greenhouse of College of Agriculture, Guangxi University, Nanning, China (22˚ ‘51’ N 108˚ ‘17’ E). Sugarcane (*Saccharum* spp.) hybrid variety ROC22 plants with 3-4 leaves of uniform growth (65 days old) was selected and treated with 150 mL of strain suspension in 6 kg of soil per pot, and control sugarcane seedlings were treated with an equal amount of sterile water. Three replications were set for each treatment, with 2 plants each pot. The soil for the pot experiment was from the greenhouse with pH 6.8, total N 1.65 g·kg^-1^, total P 0.88 g·kg^-1^, total K 18 g·kg^-1^, nitrate N 1.58 mg·kg^-1^, ammonia N 2.7 mg·kg^-1^, alkali hydrolyzed N 109 mg·kg^-1^, available P 22.0 mg·kg^-1^, inorganic P 223 mg·kg^-1^, available K 183 mg·kg^-1^, slow-releasing K 232 mg·kg^-1^, and slow-releasing potassium 232 mg·kg^-1^. Activities of polyamine oxidase (PAO) activity, reactive oxygen species (ROS)-scavenging antioxidative enzymes (superoxide dismutase (SOD) and CAT activity), and contents of PAs (Spm, Spd, Put), phytohormones (IAA, GA) and 1-aminocyclopropane-1-carboxylic synthase (ACS) in the top visible dewlap leaf (leaf +1) were analyzed at 1, 7, and 15 days after treatment (DAT), and plant height, chlorophyll and biomass were measured at 15 DAT. The PAO, Spm, Spd, Put, IAA, GA and ACS analyzed with the ELISA Kit produced by Jiangsu Meimian Co. (Jiangsu, China). SOD activity was detected by nitro blue tetrazolium (NBT). One unit of SOD activity was defined as the amount of the enzyme that inhibited the reduction of NBT by 50%. Specific activity was defined as units per mg of protein (Jain et al., 2015). The activity of CAT was analyzed by measuring the decrease in H_2_O_2_ absorbance at 240 nm (Shahid et al., 2022).

### 2.5 Co-culture of pathogen free seedlings and microorganisms

**Figure 1:**
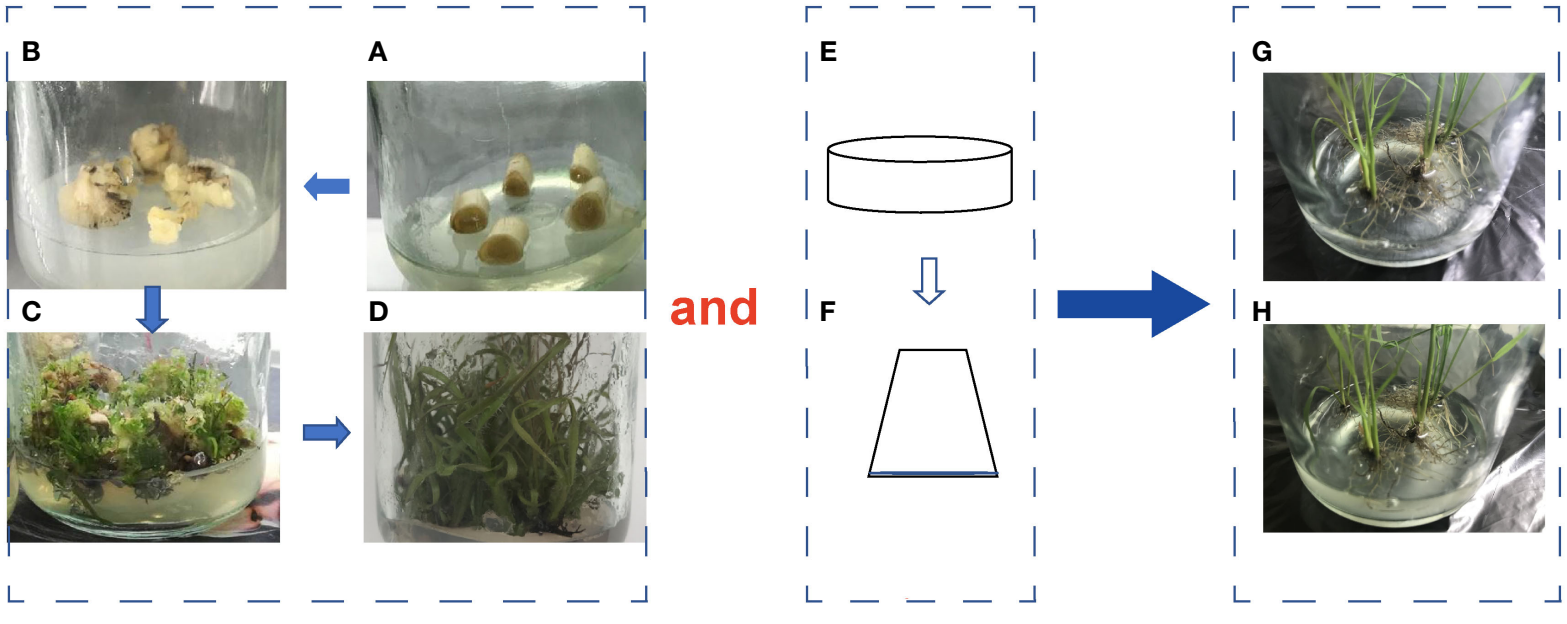
Sugarcane tissue culture and endophytic bacterial inoculation process. **(A, B)**, Differentiation; **(B, C)**, Regeneration; **(D)**, Emergence of seedlings; **(E, F)**, Bacterial strain culture; **(G)**, Uninoculated seedlings; **(H)**, Inoculated seedlings.

The detoxified micropropagated seedlings of the variety ROC22 were obtained by tissue culture. Healthy stem tops were selected and disinfected with 75% alcohol. The apical meristem tissue was obtained by cutting the top 2-4 cm of the stem tip on an clean bench, then cut into small pieces and placed on 3.0 mg·L^-1^ 2,4-dichlorophenoxyacetic acid 30 mL Murashige and Skoog (MS) solid medium to induce callus. After 40 days of dark culture, the callus was transferred to a solid MS medium autoclaved at 120°C and pH 5.8 for 20 min before being supplemented with 2.0 mg·L^-1^ N-phenylmethyl-9H-purine-6-amine and 0.1 mg·L^-1^ 1-naphthaleneacetic acid for seedling differentiation. Rooting of differentiated seedlings was induced on the solid MS medium supplemented with 3.0 mg·L^-1^ 1-Naphthaleneacetic acid. Then, the rooted seedlings were divided into single plant and transferred to liquid MS medium supplemented with 1.0 mg·L^-1^ 1-Naphthaleneaceticacid and 1.0 mgL^-1^ N-phenylmethyl-9H-purine-6-amine in an clean bench for strong seedling culture. The culture conditions were 28°C for 16 h during the day and 25°C for 8 h at night, with a light intensity of 36 μmol·m^-2^ S^-1^. The consistent seedlings were separated into single plant and placed in 30 mL of 1/10 liquid MS medium in the rate of 3 plants per bottle. 0.5 mL DX120E suspension was added, using an equal amount of sterile distilled water as control (**Figure 1**). In our pre-colonization experiments in the laboratory, it was found that the strain inoculated could be detected inside the sugarcane tissues in 1 DAT. Therefore, the transcriptome sequencing of the samples inoculated and uninoculated above-ground parts of sugarcane in 1 day after inoculation was selected.

### 2.6 Transcriptome data establishment

Total RNA was extracted from plant tissues using a Triquick reagent (Solarbio, China) and sent to Beijing Novogene Co., Ltd. for sequencing. The raw reads were filtered to remove the reads with connectors, those with an N ratio greater than 10% and those with low quality (the number of bases with Qphred =20 accounted for more than 50% of the total reads). The sequencing distribution, Q20, Q30, and GC content were used to evaluate the sequencing quality. The clean reads were spliced and analyzed using Trinity software (Grabherr et al., 2011).

### 2.7 Differential gene screening and analysis

RSEM with bowtie’s comparison was used to obtain the read count of each sample compared to each gene, which was converted to FPKM for analyzing the gene expression levels. DESeq2 (Love et al., 2014) was used for the analysis, and the screening threshold was padj < 0.05 and | log_2_FoldChange | > 1 for differential genes. Seven databases were explored for functional annotation, including NR (NCBI non-redundant protein sequences, E-value <= 1e^-5^), Nt (NCBI nucleotide sequences, E-value < = 1e^-5^), Pfam (http://pfam.sanger.ac.uk/, E-value < = 1e^-5^), KOG/COG (http://www.ncbi.nlm.nih.gov/COG/, E-value < = 1e^-5^), SwissProt (http://www.ebi.ac.uk/uniprot/, E-value < = 1e^-5^), KEGG (http://www.genome.jp/kegg/, E-value < = 1e^-5^), GO (http://www.geneontology.org/, E-value < = 1e^-5^).

### 2.8 qRT-PCR validation

Differentially expressed genes-specific primers were designed for qRT-PCR analysis (**Table 1**). Reverse transcription was performed using the kit PrimeScript™ RT Master Mix (Perfect Real Time) (Takara, Japan). The cDNA was synthesized using the reverse transcription product as a template, GAPDH as a reference gene, and fluorescence quantification was done using TB Green^®^ Premix Ex Taq™ II (Tli RNaseH Plus) (Takara, Japan) for expression detection. Relative expression was calculated by the 2^-ΔΔCt^ relative quantification method (Livak and Schmittgen, 2001). Three biological replicates were performed for each sample.

**Table 1:** Primer sequences.

Name	Sequence
GAPDH F	CTCTGCCCCAAGCAAAGATG
GAPDH R	TGTTGTGCAGCTAGCATTGGA
LAp_06H0000510 F	TGGAGGACCTGTTGACATTC
LAp_06H0000510 R	AGCAGTCTCCTGGCATAACC
LAp_06G0007890 F	GGTATTTGTGCCGTATGGAG
LAp_06G0007890 R	ACCTTATGGTTGAGGCGTAT
LAp_00065410 F	TCGGGAGGGTCTACTTCACT
LAp_00065410 R	AAGATGCGGTTGATGAGGAT
LAp_02F0007930 F	ACGGTGGTGTTCTGCGTGAG
LAp_02F0007930 R	ACGAGGGTCTTCAAATCCAA
Soffic_04G0019590-1A F	CCATGTACCTCCCGATGTTG
Soffic_04G0019590-1A R	CCATGTACCTCCCGATGTTG
Soffic_01G0001000-2D F	GCCTTCGTCGTCAACATCGG
Soffic_01G0001000-2D R	GCACCACCCTGTCCATCTCC
Sof-fic_04G0000090-3C F	AAGAGGCAGAAGGCGACCAT
Sof-fic_04G0000090-3C R	CCGAGCGAGTCAGCAAACCT
Soffic_03G0028760-5E F	GCCAAGGCTTAGCGAGTGAT
Soffic_03G0028760-5E R	CCAACCCAAACAGAAGGAGA
Soffic_01G0001480-2C F	CGGCTGTCGCTGGAGCTGAT
Soffic_01G0001480-2C R	TGGCACGGCGGGTAGTAGTT
Soffic_04G0022820-2P F	ACGACGTGAAGATCGAGACC
Soffic_04G0022820-2P R	CTAGCGTAGCCTACCCGTTT

### 2.9 Data analysis

The *Klebsiella variicola* DX120E genome data supporting the results of this article are available in the NCBI database with the accession number GCA_000812205.2. The resulted clean reads were uploaded to the NCBI database with the accession number PRJNA1010968. Microsoft (2010) Excel was used to calculate the means and standard error (SE) values. SPSS was used for the analysis of variance (One-way ANOVA with Duncan’s test, p < 0.05) and originPro (2016) for photo production.

## 3 Results

### 3.1 Analysis of genes associated with polyamine biosynthesis and transport in DX120E

**Figure 2:**
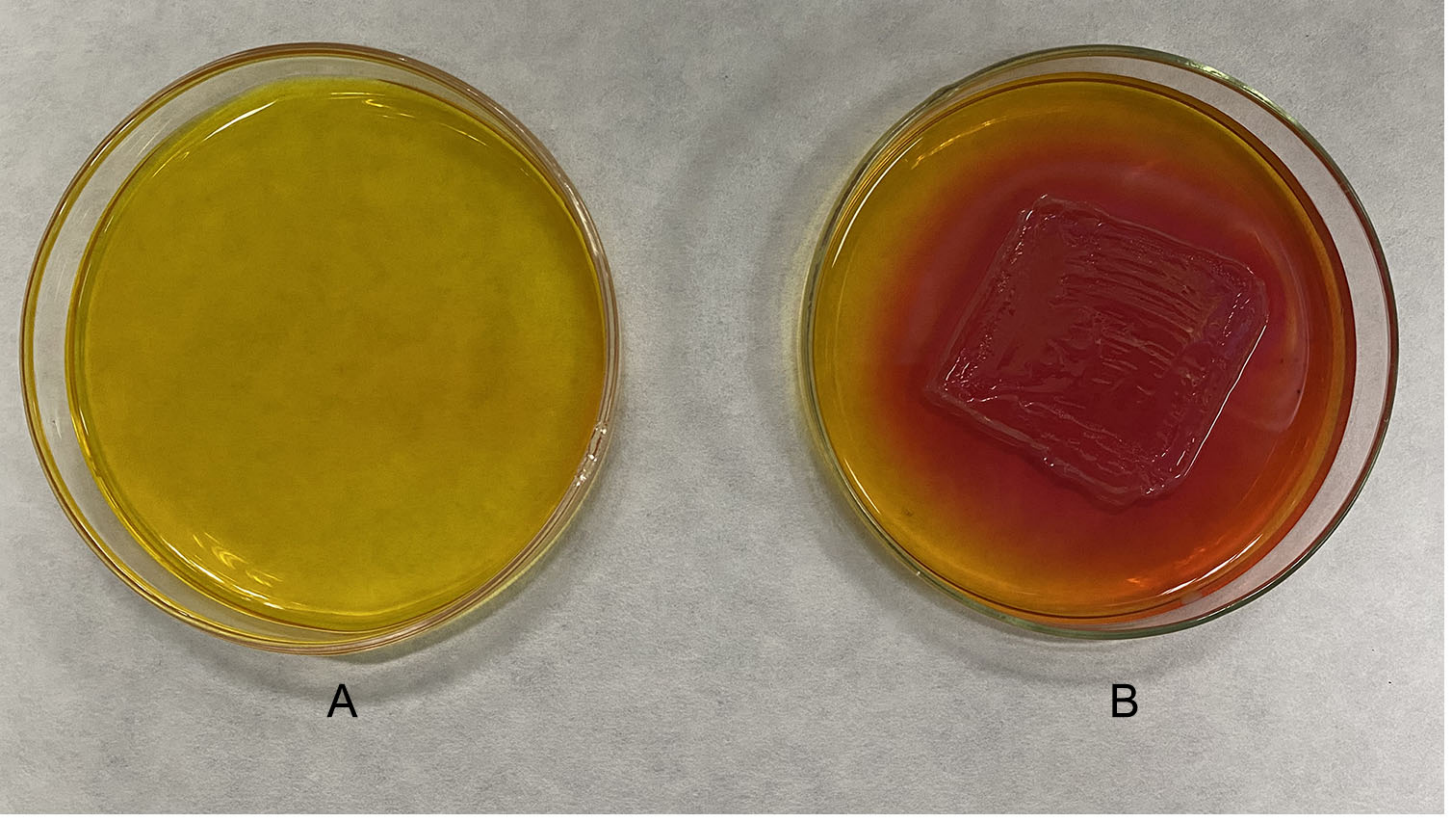
Detection of polyamine production in *Klebsiella variicola* DX120E. **(A)** Uninoculated medium. **(B)** Inoculated medium. The strain was tested on Moeller’s decarboxylase agar medium amended with L-arginine-monohydrochloride, and the change from yellow to red color of the phenol-red in medium indicated the production of putrescine.

The analysis of the strain DX120E genome showed various genes related to polyamine metabolism, including synthesis (8), and transport and degradation (31). These genes’ locus tags, specific gene types, and gene products were displayed in **Table 2**. The genes responsible for Spd biosynthesis, such as *speA* (Arginine decarboxylase, KR75_04420), *speB* (agmatinase, KR75_02455 and KR75_04415), *metK* (methionine adenosyl transferase, KR75_04430), *speC* (ornithine decarboxylase, KR75_04540), *speD* (S-adenosylmethionine decarboxylase, KR75_12420), *speE* (Spd synthase, KR75_12425), and *speG* (Spd acetyltransferase, KR75_20310) were identified in the genome of DX120E. The test of DX120E producing putrescine on Moeller’s decarboxylase agar medium plates showed that a relatively moderate to dark red halo surrounding or beneath the colonies (**Figure 2**). After culturing DX120E in an amine-free medium, the culture medium supernatant contained 208.95 ng·L^-1^ of Spm, 172.88 ng L^-1^ of Spd, and 517.15 nmol L^-1^ of Put (**Table 3**). It indicated that DX120E could produce PAs *in vitro* and could be considered as a potential Put-producing endophytic nitrogen fixing bacteria.

**Table 2:** Genes involved in polyamine transport and biosynthesis.

Pathway	Gene ID	Product	Gene	Location
Synthesis	KR75_04420	arginine decarboxylase	*speA*	754745-756643
	KR75_02455	agmatinase	*speB*	1152161-1153111
	KR75_04415	agmatinase	*speB*	756887-757797
	KR75_04430	methionine adenosyltransferase	*metK*	752671-753825
	KR75_04540	ornithine decarboxylase	*speC*	733094-735232
	KR75_12420	S-adenosylmethionine decarboxylase	*speD*	4568534-4569328
	KR75_12425	spermidine synthase	*speE*	4567649-4568509
	KR75_20310	spermidine acetyltransferase	*speG*	2908943-2909503
Degradation and transporters	KR75_04635	bifunctional glutathionylspermidine amidase/synthase	*gss*	712705-714570
	KR75_05020	putrescine aminotransferase	*ygjG*	633588-634967
	KR75_09810	Fe^3+^/spermidine/putrescine ABC transporter ATP-binding protein		5129965-5130996
	KR75_09825	spermidine/putrescine ABC transporter permease		5127048-5127905
	KR75_09940	spermidine/putrescine ABC transporter substrate-binding protein		5101970-5102389
	KR75_16570	spermidine/putrescine ABC transporter substrate-binding protein PotF	*potF*	3701527-3702639
	KR75_16575	putrescine transporter ATP-binding subunit	*potG*	3700230-3701396
	KR75_16580	putrescine ABC transporter permease PotH	*PotH*	3699266-3700219
	KR75_16585	putrescine ABC transporter permease PotI	*PotI*	3698424-3699269
	KR75_17255	gamma-glutamylputrescine oxidoreductase		3536799-3539079
	KR75_17260	aldehyde dehydrogenase PuuC	*puuC*	3535309-3536796
	KR75_17265	transcriptional regulator	*puuR*	3534431-3534988
	KR75_17270	gamma-glutamyl-gamma-aminobutyrate hydrolase	*puuD*	3533641-3534605
	KR75_17275	gamma-glutamylputrescine synthetase		3532006-3533427
	KR75_17280	Putrescine importer PuuP	*PuuP*	3530261-3531652
	KR75_17780	spermidine/putrescine ABC transporter substrate-binding protein PotD	*PotD*	3427862-3428908
	KR75_17785	spermidine/putrescine ABC transporter permease PotC	*potC*	3427080-3427865
	KR75_17790	spermidine/putrescine ABC transporter permease PotB	*potB*	3426226-3427083
	KR75_17795	putrescine/spermidine ABC transporter ATP-binding protein	*potA*	3425106-3426242
	KR75_19045	peptide ABC transporter ATP-binding protein	*sapF*	3189238-3190047
	KR75_19050	peptide ABC transporter ATP-binding protein	*sapD*	3188244-3189236
	KR75_19055	antimicrobial peptide ABC transporter permease SapC	*sapC*	3187354-3188244
	KR75_19060	putrescine ABC transporter permease SapB	*sapB*	3186402-3187367
	KR75_21485	spermidine/putrescine ABC transporter		2666914-2668014
	KR75_21725	allantoinase	*puuE*	2620490-2621422
	KR75_22430	spermidine/putrescine ABC transporter permease		2478318-2479124
	KR75_22435	spermidine/putrescine ABC transporter permease		2477399-2478328
	KR75_22440	polyamine ABC transporter ATP-binding protein		2476384-2477397
	KR75_22445	spermidine/putrescine ABC transporter substrate-binding protein		24752222476367
	KR75_23325	spermidine/putrescine ABC transporter permease		2311510-2313279
	KR75_26215	putrescine/spermidine ABC transporter	*plaP*	1725162-1726520

**Table 3:** Bacterial polyamine secretion content.

Name	Content
Spermine	208.95 ± 7.29 ng·L^-1^
Spermidine	172.88 ± 6.52 ng·L^-1^
Putrescine	517.15 ± 40.04 nmol·L^-1^

### 3.2 Effect of PAs on growth in bacteria

**Figure 3:**
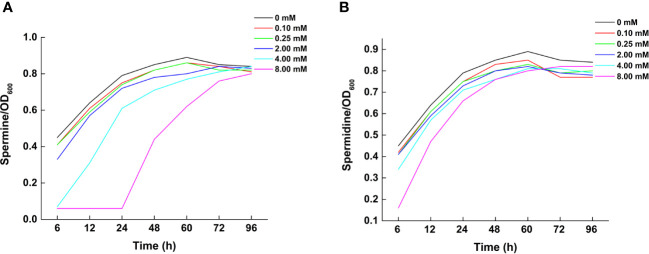
Effect of polyamine addition on growth of *Klebsiella variicola* DX120E. **(A)** Growth in response to different concentrations of exogenous spermine; **(B)** Growth in response to different concentrations of exogenous spermidine.

At Spm concentrations of 4 and 8 mM and incubation time less than 60 h, the growth of the strains were more limited compared to the other Spm concentrations. With the extension of the incubation time, the number of bacterial fluids all converged to that of the medium without exogenously added Spm. The growth of the strain without exogenous spermidine addition was consistently higher than that of the strains intervening at each concentration of spermidine. At 8 mM spermidine within 48 h of incubation time, the growth of the strain was more affected. During the 48-60 h period, the growth of strains with 8 mM spermidine was close to that of 4 mM. The effect of Spm on the strain growth was more obvious than that of Spd in the 24 h range (**Figures 3A, B**).

### 3.3 Polyamine oxidase activity and PAs contents

**Figure 4:**
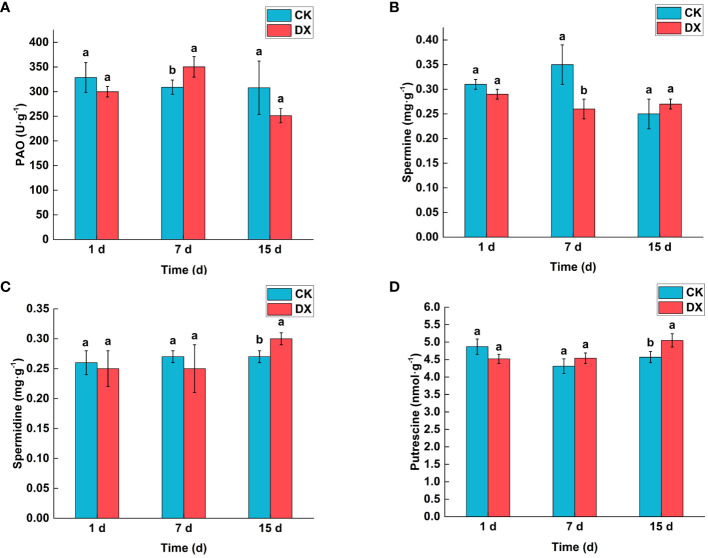
Effect of inoculation with *Klebsiella variicola* DX120E on polyamine content in sugarcane. **(A)** Polyamine oxidase; **(B)** Spermine; **(C)** Spermidine; **(D)** Putrescine; CK; Uninoculated; DX, Inoculated with *Klebsiella variicola* DX120E. The same lowercase letters above the bars indicate no significant difference between treatments in Duncan’s multiple range test, p > 0.05.

PAO activity was determined to analyze the physiological activities in sugarcane plants under DX120E inoculated and uninoculated conditions as shown in **Figure 4A**. At 1 DAT, PAO activity, Spm, Spd and Put tended to decrease but did not show significant differences. The results revealed that there was a significant (p < 0.05) increase in PAO activity (1.13 time) in sugarcane plants inoculated with DX120E compared to the control at 7 DAT. At 7 DAT, the content of Spm (**Figure 4B**) in the inoculated sugarcane leaves decreased compared to the control. After 15 days of co-incubation, all the components of PAs showed higher in the inoculated leaves compared to the control, and Spd (**Figure 4C**) and Put (**Figure 4D**) showed a significant increase over the control, respectively. These results suggested that inoculation of DX120E caused fluctuations in polyamine metabolism in sugarcane leaves.

### 3.4 Activities of ROS-scavenging antioxidative enzymes

**Figure 5:**
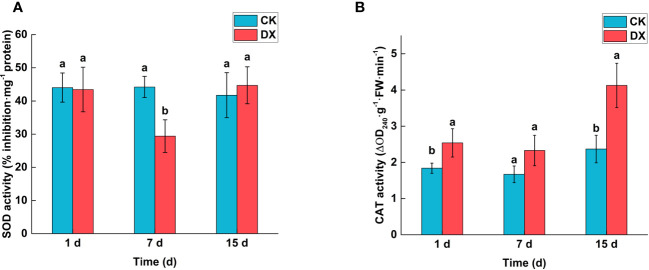
Effects of inoculation with *Klebsiella variicola* DX120E on the activeties of ROS-scavenging antioxidative enzymes in sugarcane leaves. **(A)** Superoxide dismutase (SOD) activity; **(B)** Catalase (CAT) activity. CK, Uninoculated; DX, Inoculated with *Klebsiella variicola* DX120E. The same lowercase letters above the bars indicate no significant difference between treatments in Duncan’s multiple range test, p > 0.05.

Microbial colonization in plant tissues may induce antioxidant enzymatic activities. The SOD activity showed a decreasing trend during the first seven days and a significant decrease at 7 DAT. The SOD activity in the inoculated sugarcane leaves decreased by 33.48% at 7 DAT but recovered at 15 DAT as compared to the control (**Figure 5A**). In contrast, CAT activity was increased by the DX120E colonization all the time, and was 37.95% (1 DAT) and 74.64% (15 DAT) higher than that in the control, respectively (**Figure 5B**).

### 3.5 Contents of phytohormones and ACS

**Figure 6:**
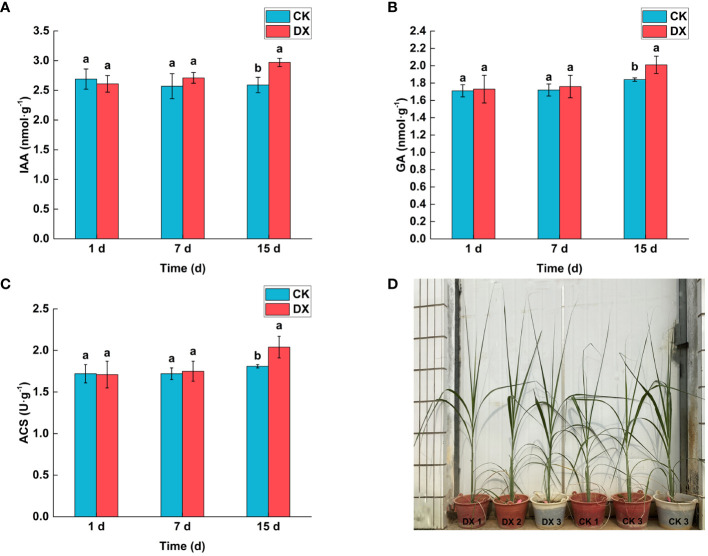
Effect of inoculation with *Klebsiella variicola* DX120E on the contents of phytohormones and 1-aminocyclopropane-1-carboxylic synthase (ACS) in sugarcane leaves. **(A)** Auxin (IAA) content; **(B)** Gibberellin (GA) content; **(C)** ACS content; **(D)** The growth of sugarcane plants. CK, Uninoculated; DX, Inoculated with *Klebsiella variicola* DX120E. The same lowercase letters above the bars indicate no significant difference between treatments in Duncan’s multiple range test, p > 0.05.

The contents of phytohormones in sugarcane leaves showed an increasing trend after inoculation with *Klebsiella variicola* DX120E, but the effect was not significant in the 7 DAT. At 15 DAT, the levels of the IAA and GA were significantly 1.15 and 1.09 times higher (p < 0.05) in the inoculated treatment than in the control respectively (**Figures 6A, B**). The trend of ACS content was consistent with both phytohormones, and that in the inoculated treatment was 1.12 times higher than that in the control at 15 DAT (**Figure 6C**).

### 3.6 Growth of sugarcane

The responses of sugarcane to endophytic bacteria inoculation were evaluated under greenhouse conditions (**Figure 6D**). The results showed that the DX120E inoculation affected SPAD, height, and shoot weight (**Table 4**). Statistical analyses showed no significant changes (p < 0.05) in the levels of SPAD, plant height, and fresh weight but a 1.45-fold significant increase in dry weight of the shoot at 15 DAT (**Table 4**).

**Table 4:** Details of *Klebsiella variicola* DX120E inoculation impacts on sugarcane growth at 15 DAT.

Treatment	Chlorophyll (SPAD)	Plant height (cm)	Fresh weight (g)	Dry weight (g)
CK	38.08 ± 0.74^a^	38.38 ± 6.84^a^	37.54 ± 6.84^a^	4.72 ± 0.50^b^
DX	40.16 ± 2.17^a^	44.6 ± 6.95^a^	40.44 ± 6.84^a^	6.88 ± 1.33^a^

CK, Uninoculated; DX, Inoculated with Klebsiella variicola DX120E. The same lowercase letters following the data indicate no significant difference between treatments in Duncan’s multiple range test, p > 0.05.

### 3.7 Transcriptomic data quality assessment

The raw fragments of sugarcane samples (L_CK and L_DX) were obtained by sequencing. Transcriptome sequencing of the six sugarcane samples generated 376, 063, 990 raw reads with over 369, 040, 004 clean reads respectively. The error rate of genes Q20, Q30 and the GC content of L_CK and L_DX were listed in **Table 5**.

**Table 5:** Summary of data output quality.

Sample	Library	Raw_ reads	Raw_ bases	Clean_ reads	Clean_ bases	Error rate	Q20	Q30	GC_content
L_CK1	XRAS230003696-2r	60381634	9.06G	59235888	8.89G	0.02	98	94.48	58.32
L_CK2	XRAS230003698-2r	60596690	9.09G	59546296	8.93G	0.03	97.8	93.97	58.17
L_CK3	XRAS230003699-2r	63788034	9.57G	62647502	9.4G	0.02	97.95	94.3	58.26
L_DX1	XRAS230003700-3r	73105722	10.97G	71851928	10.78G	0.02	98.05	94.65	57.66
L_DX2	XRAS230003702-2r	62955818	9.44G	61687988	9.25G	0.02	97.99	94.45	58.37
L_DX3	XRAS230003703-2r	55236092	8.29G	54070402	8.11G	0.02	98.02	94.5	58.26

L_CK, Sample of sugarcane uninoculated; L_DX, Sample of sugarcane inoculated with Klebsiella variicola DX120E.

### 3.8 Differential gene acquisition

**Figure 7:**
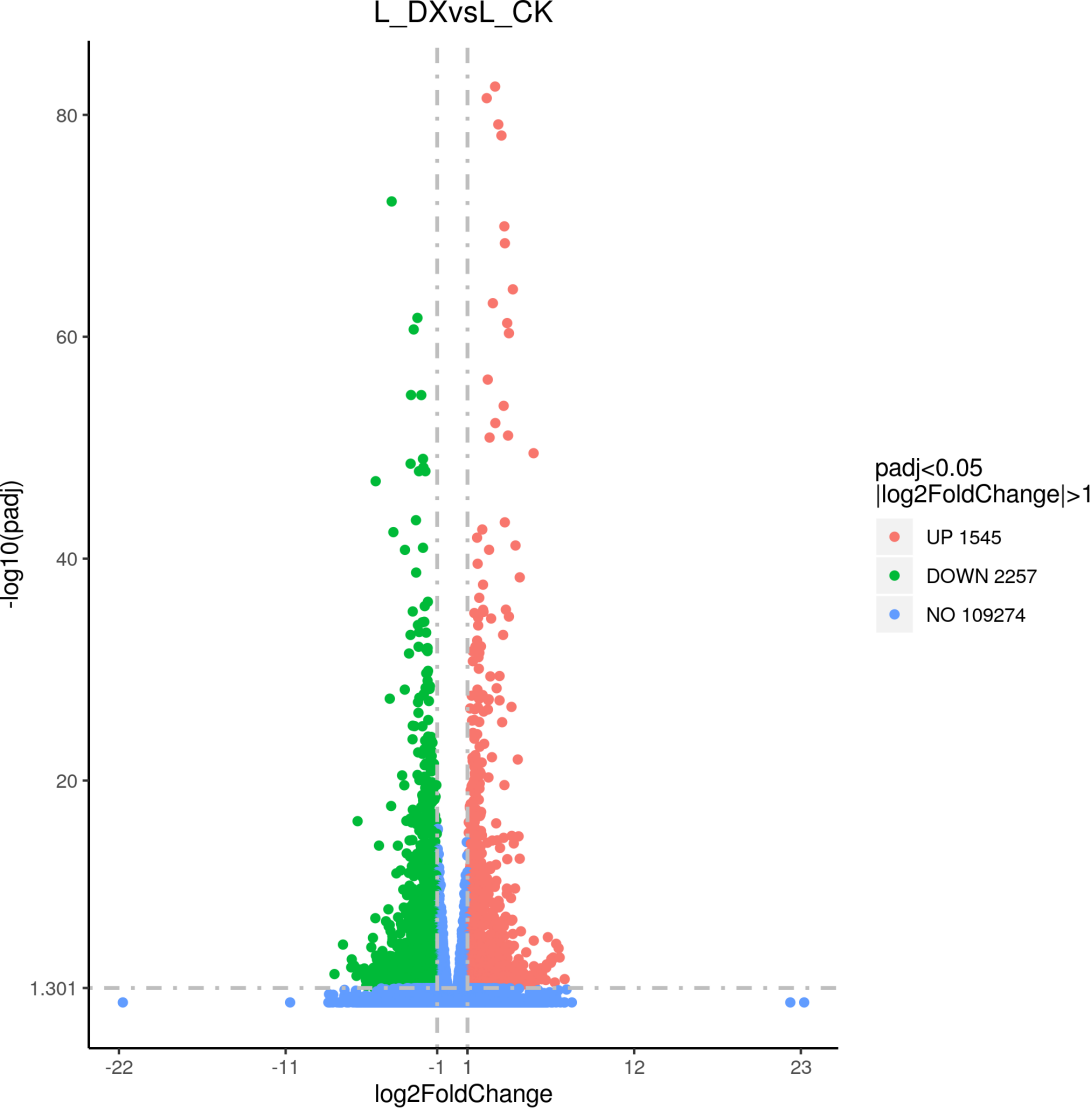
Volcano plot of differentially expressed genes (DEGs) statistics.

The differentially expressed genes (DEGs) were obtained by the comparison of the transcripts from the samples of inoculated treatment and the uninoculated control according to set screening criteria. At 1 DAT, 1545 transcripts were significantly up-regulated, and 2257 down-regulated (**Figure 7**). This trend of DEGs is considered a reflection of the interaction between sugarcane seedlings and DX120E. The high differentially expressed transcripts based on absolute fold change covered a broad spectrum of biological functions (**Supplementary Table 1**).

### 3.9 Function enrichment analysis

**Figure 8:**
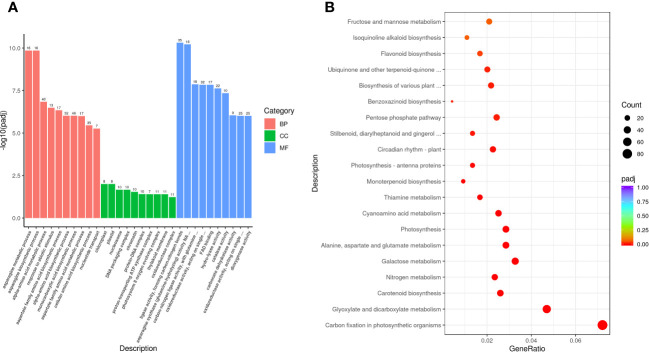
Functional enrichment analyses. **(A)** GO enrichment analysis of DEGs in L_DX vs L_CK. **(B)** KEGG pathway enrichment analysis of up-regulated DEGs in L_DX vs L_CK. The vertical axis indicates the pathways, the horizontal axis indicates the enrichment factor, the size of the dots indicates the number of genes in the pathway, and the color of the dots corresponds to the different P-adjustment ranges.

The obtained DEGs were subjected to GO and KEGG enrichment analyses, and the most top significantly enriched metabolic pathways were selected to draw the scatter plots. GO enrichment analyses showed that the DEGs received 227 functional terms categorized into biological process (BP, 132 terms), molecular function (MF, 86 terms), and cellular component (CC, 9 terms), and the top 10 of significant GO terms were used to draw the **Figure 8A**. The data in **Figure 8A** showed that 9 genes were linked with cell wall metabolic process including the terms cell wall macromolecule catabolic process (GO:0016998) and cell wall macromolecule metabolic process (GO:0044036) (**Table 6**); 13 genes were associated with the abiotic stimulus-response terms (GO:0009628) including detection of external stimulus (GO:0009581), detection of abiotic stimulus (GO:0009582), detection of stimulus (GO:0051606), and response to external stimulus (GO:0009605) (**Table 7**); 33 genes were linked with peroxidase activity (GO:0004601) and catalase activity (GO:000409). A total of 10 CAT linked genes were screened, including the catalase isozyme 1 (Soffic_10G0025850-4E, Soffic_10G0023920-1A), catalase isozyme 2 (Soffic_01G0050440-2B), catalase isozyme 3 (Soffic_04G0000090-3C, Sof-fic_04G0000300-4E, Soffic_04G0000280-1A, LAp_04D0000340, Soffic_04G0000340-2B, LAp_04H0000390, and LAp_ 04G0000230). 9 CAT genes were significantly up-regulated and Soffic_01G0050440-2B down-regulated (**Table 8**). 19 genes were involved in the hormone cluster including the terms regulation of hormone levels (GO:0010817), hormone metabolic process (GO:0042445), and cellular hormone metabolic process (GO:0034754). 6 genes including Soffic_03G0028220-6F, Soffic_03G0027230-8H, Soffic_01G0033380-1A, Lap_03B0026730, Lap_01G0024270, Lap_00008940 were associated with hormone biosynthesis (**Table 9**).

**Table 6:** Differential genes associated with cell wall.

Gene_name	Gene_chr	Gene_start	Gene_end	Gene_description	log2FoldChange (L_DXvsL_CK)
LAp_04H0020250	Chr04H	65496738	65497965	Chitinase 6	-1.30
Soffic_04G0019250-5F	Chr04F	60237228	60238450	Chitinase 6	-1.44
Soffic_04G0019300-2P	Chr04F	60372889	60373756	Chitinase 6	-1.17
Soffic_04G0019590-1A	Chr04A	56209956	56211203	Chitinase 6	-1.26
Soffic_04G0020920-4D	Chr04D	60007623	60008742	Chitinase 6	-1.07
Soffic_04G0020960-1P	Chr04D	60086613	60087491	Chitinase 6	-1.73
Soffic_04G0021240-3C	Chr04C	62591437	62592404	Chitinase 6	-1.20
Soffic_04G0021470-2B	Chr04B	62941094	62942213	Chitinase 6	-1.00
Soffic_04G0021490-6G	Chr04G	59297101	59298242	Chitinase 6	-1.17

**Table 7:** Differential genes associated with stimulus.

Gene_name	Gene_chr	Gene_start	Gene_end	Gene_description	log2FoldChange (L_DXvsL_CK)
Soffic_01G0010080-3C	Chr01C	21402412	21408061	Phytochrome C	1.03
LAp_00057990	utg004712l_251000_260999	607	4645	Phytochrome B	1.17
LAp_01B0037420	Chr01B	97458226	97465509	Phytochrome B	1.28
LAp_01C0040050	Chr01C	102675801	102682877	Phytochrome B	1.56
LAp_01F0034910	Chr01F	100127235	100130950	Phytochrome B	1.14
LAp_01F0034920	Chr01F	100132287	100135827	Phytochrome B	1.24
Soffic_01G0012760-3C	Chr01C	26769994	26781941	Phytochrome a	1.81
Soffic_01G0013340-1P	Chr01E	27364962	27368322	Phytochrome a	1.57
Soffic.04G0012570-1P	Chr04G	30767438	30768925	Dehydrin DHN1	-1.46
Soffic_04G0004970-4G	Chr04G	10747862	10749315	Dehydrin DHN1	-1.28
Soffic_04G0011700-5H	Chr04H	29131857	29133322	Dehydrin DHN1	-1.14
Soffic_04G0026540-3F	Chr04F	75985981	75987440	Dehydrin DHN1	-1.12
Soffic_04G0022820-2P	Chr04H	71778148	71779534	Dehydrin COR410	-1.71

**Table 8:** Differential genes associated with antioxidant enzyme activity.

Gene_name	Gene chr	Gene_start	Gene_end	Gene_description	log2FoldChange (L_DXvsL_CK)
Soffic_10G0025850-4E	Chr10E	72130872	72145034	Catalase isozyme 1	1.12
Soffic_10G0023920-1A	Chr10A	70548441	70551414	Catalase isozyme 1	1.61
Soffic_01G0050440-2B	Chr01B	125106009	125117013	Catalase isozyme 2	-1.03
Soffic_04G0000090-3C	Chr04C	665957	668108	Catalase isozyme 3	2.42
Soffic_04G0000300-4E	Chr04E	957504	959988	Catalase isozyme 3	1.52
Soffic_04G0000280-1A	Chr04A	870913	873135	Catalase isozyme 3	2.00
LAp_04D0000340	Chr04D	874684	876920	Catalase isozyme 3	2.07
Soffic_04G0000340-2B	Chr04B	994264	996505	Catalase isozyme 3	1.53
LAp_04H0000390	Chr04H	1067831	1070074	Catalase isozyme 3	1.16
LAp_04G0000230	Chr04G	576216	578443	Catalase isozyme 3	1.04
LAp_01H0026250	Chr01H	75241540	75243819	Peroxidase 15	1.24
Soffic_03G0010230-1A	Chr03A	24214834	24217875	Peroxidase 24	1.55
Soffic_03G0010930-3D	Chr03D	26581025	26583930	Peroxidase 24	1.09
Soffic_03G0028760-5E	Chr03E	74567949	74569687	Peroxidase 3	-1.71
Soffic_03G0027780-1A	Chr03A	73030981	73032685	Peroxidase 3	-1.28
Soffic_03G0027220-2B	Chr03B	75473831	75475528	Peroxidase 3	-2.18
Soffic_03G0027340-3C	Chr03C	71901433	71903191	Peroxidase 3	-1.37
Soffic_03G0028300-4D	Chr03D	74630436	74632134	Peroxidase 3	-2.71
Soffic_05G0001820-1P	Chr05B	4555039	4557181	Peroxidase 4	-3.29
Soffic_05G0001970-3C	Chr05C	4793451	4795729	Peroxidase 4	-2.14
Soffic_06G0025150-2C	Chr06C	66437224	66438546	Peroxidase 42	1.71
Soffic_07G0000460-2B	Chr07B	1519245	1520567	Peroxidase 47	6.51
Soffic_07G0000800-4E	Chr07E	2102047	2103401	Peroxidase 47	1.78
Soffic_09G0005020-3C	Chr09C	13331390	13333282	Peroxidase 5	1.29
LAp_09E0020550	Chr09E	65228689	65229684	Peroxidase 5	-1.36
Soffic_07G0017650-2B	Chr07B	56936834	56941313	Peroxidase 50	-3.16
Soffic_10G0013130-1A	Chr10A	42575770	42576949	Peroxidase 52	1.21
Soffic_04G0008620-5F	Chr04F	20741981	20743698	Peroxidase 52	1.68
Soffic_09G0014040-2B	Chr09B	47739113	47740428	Peroxidase 54	-1.13
Soffic_03G0002070-3F	Chr03F	4212111	4213321	Peroxidase 67	2.01
Soffic_04G0009660-5H	Chr04H	23258700	23259844	Peroxidase 70	2.01
Soffic_09G0018850-3C	Chr09C	57266557	57275184	Respiratory burst oxidase homolog protein F	-1.18
LAp_06C0017920	Chr06C	53777593	53780236	Thylakoid lumenal 29 kDa protein, chloroplastic	-3.11

**Table 9:** Differential genes associated with hormones.

Gene_name	Gene_chr	Gene_start	Gene_end	Gene_description	log2Fold Change (L_DXvsL_CK)
Soffic_01G0033380-1A	Chr01A	82467869	82469177	Allene oxide cyclase, chloroplastic	-1.40
LAp_01G0024270	Chr01G	77584890	77586180	Allene oxide cyclase, chloroplastic	-1.15
Soffic_10G0000890-1A	Chr10A	2921627	2925975	Cytochrome P450 88A1	2.67
Soffic_10G0000930-2B	Chr10B	3032280	3036699	Cytochrome P450 88A1	2.13
Soffic_03G0028220-6F	Chr03F	76748549	76752574	Cytokinin dehydrogenase 5	-1.04
Soffic_03G0027230-8H	Chr03H	72042308	72046551	Cytokinin dehydrogenase 5	-1.11
LAp_03B0026730	Chr03B	74412918	74417154	Cytokinin dehydrogenase 5	-2.34
LAp_00008940	utg000345l_506999_664533	101601	106496	Cytokinin dehydrogenase 8	-1.58
Soffic_01G0001000-2D	Chr01D	2731919	2733016	Gibberellin 20 oxidase 1-B	2.56
Soffic_01G0001480-2C	Chr01C	3843666	3844793	Gibberellin 20 oxidase 1-B	2.27
Soffic_01G0000850-3E	Chr01E	2623485	2624582	Gibberellin 20 oxidase 1-B	2.20
Soffic_01G0000870-1A	Chr01A	2383960	2385090	Gibberellin 20 oxidase 1-B	1.30
Soffic_03G0025020-4G	Chr03G	69939099	69940996	Gibberellin 2-beta-dioxygenase 3	-1.16
Soffic_06G0016400-1A	Chr06A	48796850	48803340	Aldehyde dehydrogenase family 3 member F1	2.13

The DEGs were enriched to a total of 97 KEGG metabolic pathways and the top 20 shown in **Figure 8B**. The KEGG enrichment analysis results showed that Soffic_06G0016400-1A (2.13 time) identified at tryptophan metabolism (sbi00380) is a gene for aldehyde dehydrogenase (NAD+) [EC:1.2.1.3], an intermediate enzyme associated with the IAA generation. In sbi00330 (Arginine and proline metabolism), four genes were related to polyamine metabolism, including LAp_06H0000510 (arginase, 1.67 time), LAp_06G0007890 (Polyamine oxidase 3 - 1.36 time), LAp_02F0007930 (Spermidine synthase 1, 1.41 time), and LAp_00065410 (Polyamine oxidase 6, 1.47 time) (**Table 10**). It is suggested these genes were associated with pathways critical for regulating the colonization of endophytic nitrogen fixing bacteria in sugarcane seedlings (**Figure 8B**). In sbi00904 (diterpenoid biosynthesis), the genes including Soffic_06G0016400-1A, Soffic_01G0001000-2D, Soffic_01G0001480-2C, Soffic_01G0000850-3E, Soffic_01G0000870-1A, Soffic_10G0000890-1A, Soffic_10G0000930-2B, and Soffic_03G0025020-4G, were involved in GA synthesis.

**Table 10:** Differential genes associated with polyamine metabolism.

Gene_name	Gene_chr	Gene_start	Gene_end	Gene_description	log2Fold Change (L_DXvsL_CK)
LAp_06H0000510	Chr06H	2347302	2351438	Arginase 1, mitochondrial	1.67
LAp_06G0007890	Chr06G	30873758	30878908	Polyamine oxidase 3	-1.36
LAp_02F0007930	Chr02F	25973862	25977608	Spermidine synthase 1	1.41
LAp_00065410	utg006656l_1_74999	46743	51389	Polyamine oxidase 6	1.47

### 3.10 qRT-PCR

**Figure 9:**
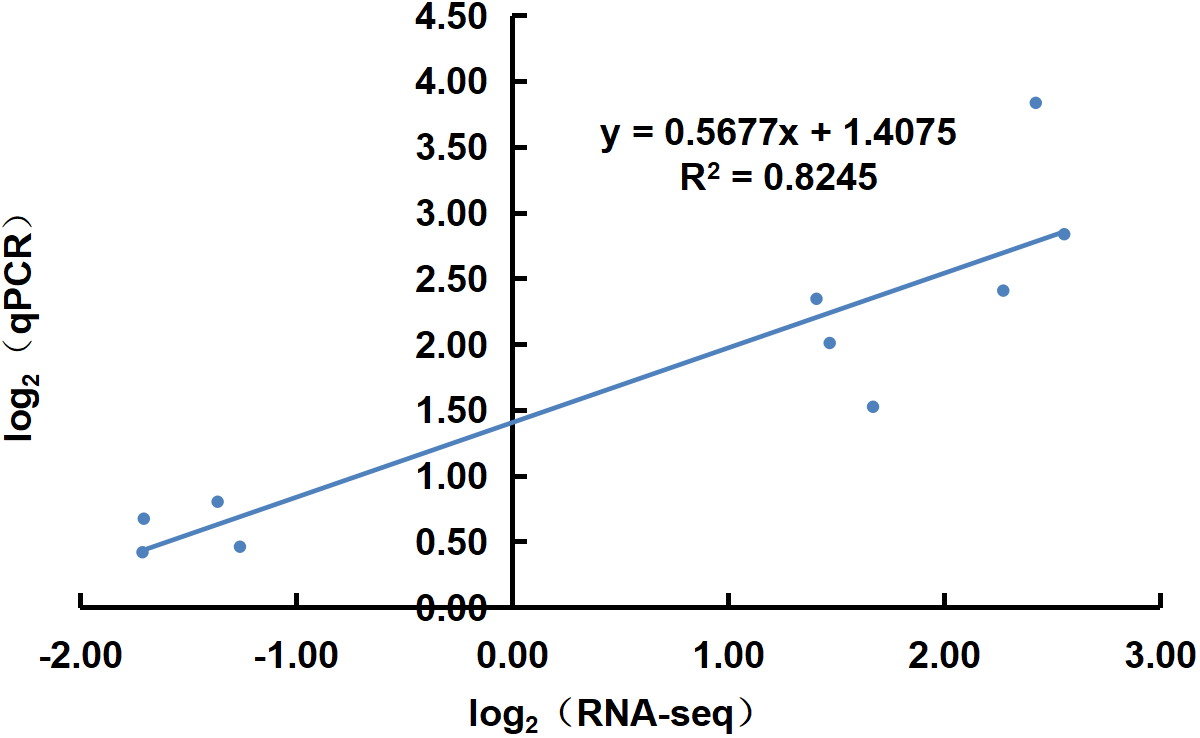
Correlation plot of qRT-PCR and RNA sequencing results.

Ten transcripts were randomly selected for qRT-PCR quantification with three experimental replicates per sample. Correlation analysis with the transcriptome data showed that the qRT-PCR results supported the RNA-Seq quantification results (R^2^ = 0.8245) (**Figure 9**).

## 4 Discussion

Nitrogen is one of the main limiting factors to plant growth. Research on sugarcane endophytic bacteria has been mostly focused on biological nitrogen fixing bacteria (Trivedi et al., 2021). Bacteria of the genus *Klebsiella* belonging to the family *Enterobacteriaceae* stimulated plant growth via an array of direct and indirect mechanisms that are of increasing biotechnological interest due to their potential bio-inoculants for plant growth (Lin et al., 2019; Medina-Cordoba et al., 2021). Carolina et al. (2018) reported that the N-containing molecules, amino acids, and mainly the excreted PAs could be derived when bacteria can fix nitrogen and release these compounds into either the rhizosphere or inside the plant. Genomic analysis of the endophytic nitrogen fixing bacteria DX120E revealed eight genes related to polyamine synthesis and 31 related to the transport and degradation. Furthermore, the strain carried all necessary genes (*metK* and *spe*ABDE) to produce the polyamine Spd. Several genes, including *potA*, *potB*, *potC*, *potD*, *potF*, *potG*, *potH*, *potI*, and *plaP* associated with Spd and Put transport were also identified. Meanwhile, it was identified that DX120E was capable of producing PAs (**Figure 2**) and three types of PAs (Spm, Spd and Put) were detected in the amine-free medium (**Table 3**) in the present study. The effects of Spm up to 2 mM and Spd up to 4 mM in the medium on the growth of nitrogen fixing bacteria DX120E were not significant, and the strain growth at each concentration of polyamine inclined to that of the medium without added polyamine at the late stage of growth (**Figure 3**). It indicated that DX120E was capable of adapting to environmental changes by polyamine metabolic pathway. PGPB bacteria carry genes that bestow favorable qualities on their host plants, and they can function as biofertilizers and bioprotectants, resulting in considerable improvements in production and enhanced tolerance to both biotic and abiotic challenges in plants (Li et al., 2021; Singh et al., 2021).

PAO converts Spm or thermos ermine into Spd and Spd into putrescine (Gerlin et al., 2021). The PAO activities in sugarcane leaves co-incubated with DX120E showed an increasing trend and was 1.13 times significantly higher than the non-inoculated control at 7 DAT, however, the enzyme activity was reduced at 15 DAT (**Figure 4A**). Spm, Spd and Put did not show significant differences on the first day but fluctuated at 7 DAT and all showed an upward trend at 15 DAT (**Figures 4B–D**). Wei et al. (2014) reported that the number of nitrogen-fixing bacteria DX120E in sugarcane leaves peaked at 2 DAT or a minimum at 15 DAT, and later started gradually increase. It is speculated that the PAs fluctuation may be caused by microbial invasion in the plant, which leads to effector-triggered immunity (Taheri, 2022), but the amount of polyamine in the plant does not inhibit the growth of the bacterium. The metabolome of cultivated wheat plants inoculated with two endophytes (*Acremonium sclerotigenum* and *Sarocladium implicatum)* revealed that the levels of metabolites (asparagine and glutamate) were significantly altered between the inoculated and non-inoculated plants and involved in the production of osmolytes such as proline and polyamines (Mishra et al., 2022).

Plant associated beneficial bacteria are known to mitigate plant diseases either directly through microbial antagonism or indirectly through plant induction of systemic resistance (ISR) (Agisha et al., 2017). In this study, ROS-scavenging antioxidative enzymes (SOD and CAT) related to plant immunity were detected in leaves of sugarcane variety ROC22. SOD enzyme activity showed a decreasing and then increasing trend in the inoculated sugarcane leaves and was significantly lower than the untreated leaves at 7 DAT (**Figure 5A**). CAT activity showed a gradual increasing trend and was significantly higher than the control at 1 DAT and 15 DAT (**Figure 5B**). The ROS-scavenging antioxidative enzymes changes indicate that DX120E triggers the plant defense system. The interaction of numerous beneficial bacteria with their host plants has been widely investigated, and resistance induction has been recorded in various crops (Mayak et al., 2004; Agisha et al., 2017).

Phytohormones levels and ACS in sugarcane leaves changed after inoculation of DX120E. The contents of IAA, GA, and ACS in the inoculated sugarcane leaves indicated a gradual and significant increase compared to the un-inoculated sugarcane. Several authors attributed the increase in root development of plants inoculated with endophytic bacteria to the release of auxin by the bacteria (Carolina et al., 2018). Straub et al. (2013) reported that endophytic bacteria *H. frisingense* affected the signaling of plant hormones, namely ethylene signaling, in root growth. Plant ethylene precursor 1-aminocyclopropane-1-carboxylic acid (ACC) showed various roles in microorganism’s developmental processes along with plant growth promotion abilities (Nascimento et al., 2014; Vanderstraeten et al., 2019). Nitrogen fixing capacity and hormone regulation makes the nitrogen fixing bacterium DX120E become a potential promotion strain.

In this study, transcriptomic analysis of sugarcane variety ROC22 under bacterial colonization compared to the control found 3802 genes activated on plant-bacteria interaction. The obtained DEGs received 227 GO terms (**Figure 8A**) and 97 KEGG pathways (**Figure 8B**) on enrichment analysis. Two terms (GO:0016998, GO:0044036) were associated with the cell wall, 5 terms (GO:0009628, GO:0009581, GO:0009582, GO:0051606, GO:0009605) connected with stimulus-response and 2 terms (GO:0004601, GO. 0004096) linked with peroxidase activity. The identified KEGG pathways were linked to tryptophan metabolism, diterpenoid biosynthesis (hormones), and arginine and proline metabolism (polyamine metabolism). Eight DEGs associated with phytochrome a/b/c were significantly up-regulated, whereas 9 DEGs related to chitinase 6 (cell wall) and 5 DEGs linked to dehydrin DHN1/COR410 were down-regulated (**Table 7**). Twenty nine DEGs associated with antioxidant enzyme activity (**Table 8**) particularly included catalase isozyme 1/2/3, peroxidase 3/4/5/15/24/42/47/50/52/54/67/70, respiratory burst oxidase homolog protein F, thylakoid lumenal 29 kDa protein, and chloroplastic. Catalase isozyme 1/3 was significantly up-regulated, while catalase isozyme 2 was down-regulated in treatments compared to control. Peroxidase 15/24/42/47/52/67/70 was up-regulated, and peroxidase 3/450/54 was down-regulated in the DX120E inoculated treatment. The respiratory burst oxidase homolog protein F associated with the MAPK signaling pathway was down-regulated in treatment. Fifteen DEGs involved in hormone regulation included 1 DEG associated with IAA synthesis (Soffic_06G0016400-1A: Aldehyde dehydrogenase family 3 member F1), 8 DEGs with GA synthesis (LAp_03B0026730, LAp_ 00008940, Soffic_01G0001000-2D, Sof-fic_01G0001480-2C, Soffic_01G0000850-3E, Soffic_01G0000870-1A, Sof-fic_03G0025020-4G, Soffic_ 06G0016400-1A), 2 DEGs with Jasmonic acid synthesis (Soffic_10G0000890-1A, Soffic_03G0028220-6F) and 4 DEGs with cytokinin (Soffic_01G0033380-1A, LAp_ 01G0024270, Soffic_10G0000930-2B, Soffic_03G0027230-8H) (**Table 9**). For the 4 key DEGs associated with polyamine metabolism, LAp_06H0000510, LAp_02F0007930, and LAp_00065410 were up-regulated while LAp_06G0007890 was down-regulated (**Table 10**). The RNA-seq analyses results revealed remarkable beneficial plant–bacteria interactions. The functional genes of cell wall and peroxidase activity suggested that the endophytic bacteria DX120E stimulated the immune response of sugarcane. Plants accumulate osmolyte compounds in response to biotic stresses. Major cellular osmolytes, including proline, glycine betaine, and PAs, are found in plants and bacteria (Gholizadeh and Mirzaghaderi, 2020). PAs and PAO have been proven to differ significantly in a micro-sugarcane intercropping system. The biosynthesis of polyamines requires ornithine, arginine, and glutamate as precursors, therefore, they are strongly regulated together (Chen et al., 2021). PAs can be oxidized by copper-containing diamine oxidases and polyamine oxidases (PAOs). PAOs are divided into two major groups. The first group catalyzes Spd and Spm to produce 1,3-diamino propane (DAP), H_2_O_2_ (Asghari et al., 2020), and N-3-aminopropyl-4-amino butanal or 4-amino butanal, which is referred to as the terminal catabolism pathway (Sagor et al., 2021). The second group is involved in the back conversion of Spm to Spd and Spd to Put. Common to all PAO reactions is the production of H_2_O_2_. Cell wall reconstitution, a crucial step in regeneration, relies on H_2_O_2_-dependent peroxidase activity (Papadakis and Roubelakis-Angelakis, 2005). A previous study revealed that Spd and Spm stimulate elongation growth and reduce membrane damage in wheat (Ebeed et al., 2017). PAs reduced the accumulation of O_2_ but not that of H_2_O_2_. It was assumed that PAOs are involved in plants’ response to cell development and biotic stress (Hao et al., 2018). Cellular PAs can act as endogenous antioxidant molecules. In this study, the expression of the CAT and peroxidase genes and SOD has decreased and SOD activity of leaves treatment by DX120E were lower compared to the control, reflecting the significant regulation of ROS-scavenging antioxidative enzymes by DX120E.

Similarly, Nassar et al. (2003) found that polyamine-producing *S. griseoluteus* promoted the growth of bean plants under greenhouse conditions by increasing the endogenous levels of Put, Spd, Spm, IAA, and GA. Soffic_06G0016400-1A is a gene associated with aldehyde dehydrogenase family 3 member F1, which is related to the synthesis of IAA (Dhungana and Itoh, 2019; Ul Hassan and Bano, 2019; Zhang et al., 2019). The up-regulation of cytochrome P450 88A1 and gibberellin 20 oxidase 1-B flanked the up-regulation of GA content in the microbe-sugarcane combination. Polyamine is also an intermediate signaling molecule in ACC signaling. Some beneficial microbes are ethylene regulators by affecting ACC deaminase, which reduces ethylene levels while promoting plant growth and defense (Kong et al., 2022). ACC deaminase lowers ethylene levels by converting ACC precursor into α-ketobutyrate and ammonia (Mukherjee et al., 2020). It is thought this situation occurs in an environment where the endophytic bacteria remain relatively stable with the sugarcane system, however, further verification is necessary. PAs are generally considered a class of plant growth regulators which mediate phytohormone effects or independently act as signaling molecules (El-Tarabily et al., 2020).

**Figure 10:**
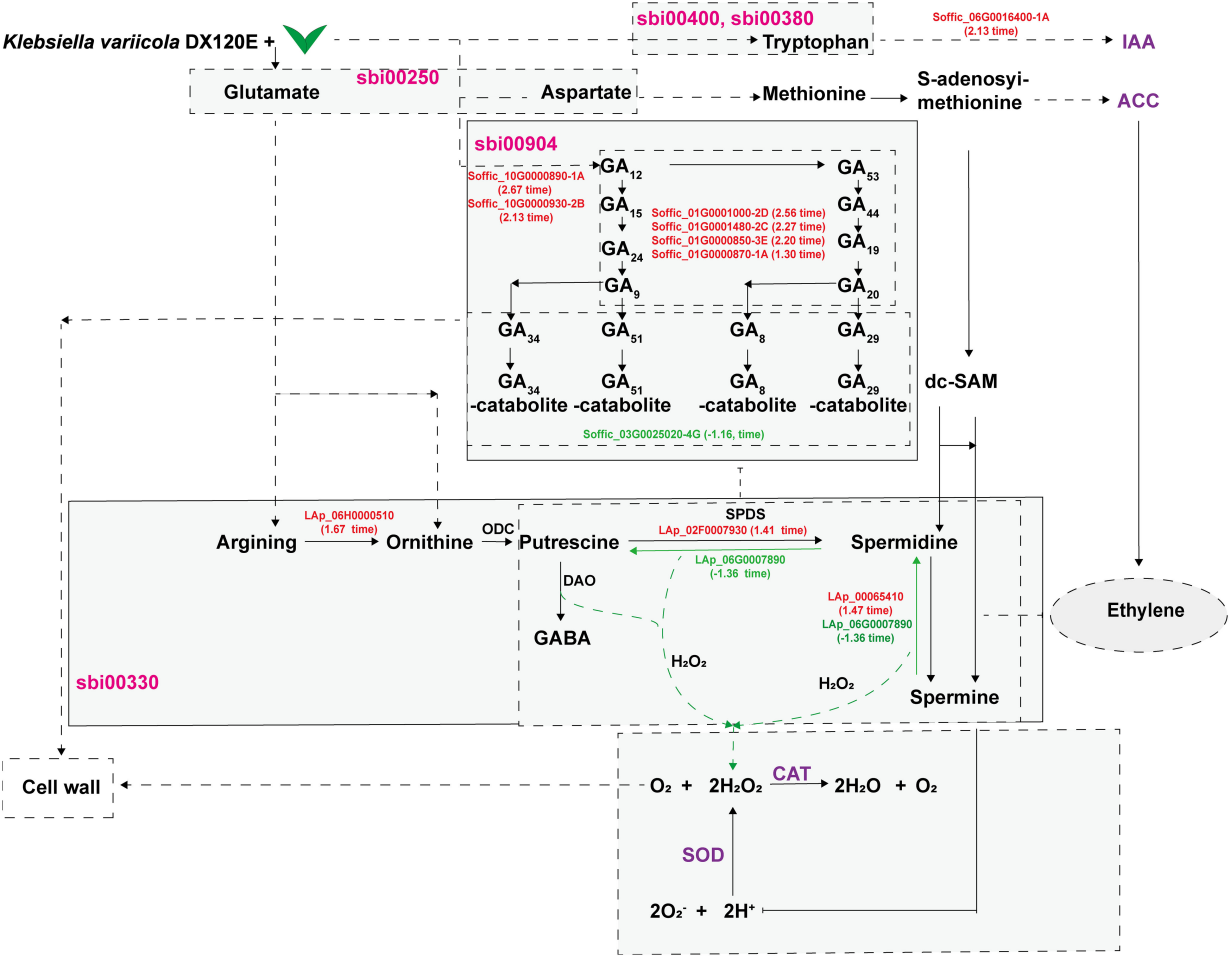
DX120E inoculation effect on sugarcane polyamine metabolism and relevant pathways.

Based on the data obtained in this study, a model diagram centered around polyamine metabolism showing the effects of DX120E inoculation on sugarcane was drawn (**Figure 10**). In the model, the secretion of PAs or biological nitrogen fixation stimulates fluctuations in amino acid metabolism, polyamine metabolism, ROS-scavenging antioxidative enzymes, and phytohormones in sugarcane variety ROC22. Amine oxidases catalyze the degradation of PAs, producing H_2_O_2_ and ammonia. Then, H_2_O_2_ and PAs directly or indirectly affect the formation of reactive oxygen species and cell walls in plant tissues or promote plant growth by regulating the synthesis of hormones. Polyamines have multiple functions, such as homeostasis, influencing responses to biotic stresses, phytohormone metabolism, and promoting plant growth. The available data have demonstrated that the fluctuation of polyamine metabolism is involved in the interaction of associated nitrogen fixation system as associated with plant promotion.

## 5 Conclusion

PGPB, with nitrogen fixing ability, are a valuable source of nitrogen for sustainable crop production. This study found that endophytic nitrogen-fixing bacteria *Klebsiella variícola* DX120E isolated from sugarcane variety ROC22 produced polyamines *in vitro* culture medium, and 39 DEGs were related to the transport and degradation of polyamine. The activities of PAO and ROS-scavenging antioxidative enzymes, and the contents of polyamine, phytohormones, and ACS in leaves of sugarcane were strongly involved in response to endophytic bacteria DX120E. Transcriptomic analysis found that 73 DEGs obtained from the leaves of sugarcane colonized by DX120E were related to the cell wall, stimulus-response, peroxidase activity, tryptophan metabolism and diterpenoid biosynthesis. Genetic and molecular approaches would assist in further understanding the acting mechanisms of nitrogen-fixing microorganisms and the roles of PAs in microbe-plant interaction, which could be valuable to boost future research for the effective application of the bacterial strain in crop improvement.

## Data availability statement

The datasets presented in this study can be found in online repositories. The names of the repository/repositories and accession number(s) can be found below: BioProject, PRJNA1010968.

## Author contributions

YQ: Writing – original draft. QK: Writing – review & editing. J-WY: Investigation, Writing – original draft. Y-YW: Investigation, Writing – original draft. Y-FP: Investigation, Writing – original draft. YH: Investigation, Writing – original draft. J-LW: Investigation, Writing – original draft. D-JG: Investigation, Writing – original draft. Y-RL: Supervision, Writing – review & editing. D-FD: Supervision, Writing – review & editing. Y-XX: Funding acquisition, Project administration, Supervision, Writing – review & editing.
